# The Effects of Sevoflurane vs. Propofol for Inflammatory Responses in Patients Undergoing Lung Resection: A Meta-Analysis of Randomized Controlled Trials

**DOI:** 10.3389/fsurg.2021.692734

**Published:** 2021-07-02

**Authors:** Jing-Li Yuan, Kang Kang, Bing Li, Jie Lu, Meng-Rong Miao, Xia Kang, Jia-Qiang Zhang, Wei Zhang

**Affiliations:** ^1^Department of Anesthesiology and Perioperative Medicine, Henan University People's Hospital, Henan Provincial People's Hospital, Zhengzhou, China; ^2^Department of Health Statistics, School of Public Health, Zhengzhou University, Zhengzhou, China; ^3^Department of Anesthesiology and Perioperative Medicine, Zhengzhou University People's Hospital, Henan Provincial People's Hospital, Zhengzhou, China

**Keywords:** sevoflurane, propofol, OLV, inflammatory response, meta-analysis

## Abstract

**Objective:** Inflammatory cytokines are increased during one-lung ventilation in patients undergoing lung resection, and this increase can be fatal. Propofol and sevoflurane are the main anesthetics used for these patients. Unfortunately, there is no consensus on the best choice of an anesthetic agent concerning an inflammatory response in patients undergoing lung resection. This meta-analysis aimed to compare the effects of propofol and sevoflurane on the inflammatory response in patients undergoing lung resection.

**Methods:** We searched electronic databases to identify randomized controlled trials comparing the effects of different anesthetics (sevoflurane vs. propofol) on the inflammatory response. The primary outcome concerned the concentration of systemic inflammatory cytokines. The secondary outcomes concerned the concentrations of inflammatory cytokines in the bronchoalveolar lavage (BAL) fluid from the dependent and independent lung. Random effects analysis of the meta-analyses were performed to synthesize the evidence and to assess the concentrations of inflammatory factors in the sevoflurane and propofol groups.

**Results:** Eight trials involving 488 participants undergoing lung resection with one-lung ventilation were included. There was no significant difference in the concentrations of systemic interleukin (IL)-6, IL-10, or tumor necrosis factor α between the sevoflurane and propofol groups. Compared with the propofol group, BAL levels of IL-6 in the dependent ventilated lung were decreased in the sevoflurane group (three trials, 256 participants; standardized mean difference [SMD], −0.51; 95% confidence interval [CI], −0.90 to −0.11; *p* = 0.01; *I*^2^ = 46%). The BAL levels of IL-6 in the independent ventilated lung were also decreased by sevoflurane (four trials, 362 participants; SMD, −0.70; 95% [CI], −0.93 to −0.47; *p* < 0.00001; *I*^2^ = 0%).

**Conclusions:** There was no difference in the systemic inflammatory response between the sevoflurane and propofol groups. However, compared with propofol, sevoflurane can reduce the local alveolar inflammatory response. Additional research is necessary to confirm whether the inflammatory response is direct or indirect.

## Introduction

The incidence of lung cancer is currently increasing and lung resection is believed to be an important treatment for this malignancy (1). One-lung ventilation (OLV) is typically required during lung resection. The inflammatory response is activated intraoperatively; these responses are subsequently induced (2, 3). The primary cytokines released after surgery are interleukin (IL)-6 and tumor necrosis factor α (TNF-α). The initial response is the release of TNF-α by activated macrophages and monocytes in damaged tissue. This stimulates the production and release of more cytokines, particularly IL-6, which is the main cytokine in the acute phase response and is responsible for inducing systemic changes (4). Animal experiments showed that re-expansion of the lung after a short period of OLV caused the release of alveolar pro-inflammatory cytokines (5). The results from a human study suggested that intraoperative inflammatory cytokines were increased in patients undergoing thoracic surgery (5), and temporary lung collapse and surgical operations could enhance the expression of inflammatory mediators (3, 6, 7).

Cytokines play an important role in the inflammatory response to surgery and trauma (4); however, an excessive inflammatory response can lead to serious complications. The recruitment of neutrophils by pro-inflammatory cytokines leads to endothelial cell damage. These responses typically occur before systemic inflammatory response syndrome or acute respiratory distress syndrome (8). Although some cytokine responses may maintain homeostasis in patients undergoing cardiothoracic surgery, catabolic states induced by acute mediators can be severe or fatal (9). Moreover, inflammatory responses induced by OLV can be life-threatening in severe cases (10–12). Researchers suggested that increased inflammatory cytokines may be associated with pulmonary complications after pneumonectomy (13). Therefore, inhibiting these over-expressed inflammatory responses will help improve the clinical outcomes.

Propofol and sevoflurane are the main anesthetics used worldwide in patients undergoing lung resection. The differences between the effects of propofol and sevoflurane on the inflammatory response are controversial. A study by de la Gala et al. (14) showed that the inflammatory response in the lungs (and in the entire body) could be reduced by sevoflurane during pneumonectomy. However, a study by Schilling (3) found that sevoflurane could inhibit local alveolar inflammation but not the systemic inflammatory response. Furthermore, results in (15) indicate that the perioperative inflammatory reaction to propofol was lower compared with sevoflurane, and lung injury using propofol was lower compared with sevoflurane. Lee (8) found that sevoflurane could reduce the systemic increase of IL-6 at the end of surgery. Based on these results, the effects of sevoflurane and propofol on inflammatory responses have not been consistently determined.

The purpose of this meta-analysis was to compare the effects of sevoflurane and propofol on the inflammatory response in patients undergoing lung resection.

## Materials and Methods

A meta-analyses was conducted following the Cochrane Handbook for systemic Reviews of Interventions (16) and was reported in compliance with the Preferred Reporting Items for systemic Reviews and Meta-Analyses (PRISMA) statement (17). It was prospectively registered with the PROSPERO registry (CRD42020204577).

### Eligibility Criteria

The eligibility criteria of the current study were as follows.

- Types of trials: Randomized controlled trials.
- Participants: Patients undergoing lung resection with OLV.
- Intervention types: Anesthesia maintained by propofol or sevoflurane.
- Types of outcome measures: The primary outcome was the concentration of systemic inflammatory cytokines. The secondary outcomes were the concentrations of inflammatory cytokines in the bronchoalveolar lavage (BAL) fluid from the dependent and independent lung.

### Search Strategy

One reviewer (JL Y) searched PubMed, Embase, and the Cochrane Central Register of Controlled Trials through May 1, 2020, without any restrictions. A controlled vocabulary (the Medical Subject Headings indexing system for PubMed and the Embase subject headings indexing system for Embase) and keywords were used. Search terms included those related to OLV, sevoflurane, propofol, and their variants. The complete search strategy is available in Appendix 1 of this paper. Two reviewers (JL Y and K K) manually-checked the reference lists of eligible trials and existing reviews.

### Trial Selection

Once the records were imported into EndNote reference management software (Clarivate Analytics), duplicate records were removed. Two reviewers (JL Y and B L) screened the titles and abstracts for relevance and labeled records as included, excluded, or uncertain. In the case of uncertainty, the full-text articles were retrieved to assess their eligibility. Disagreements were resolved by discussion with other reviewers.

### Data Extraction

Two reviewers (JL Y and K K) independently extracted the data using a standardized form. We collected information on trial characteristics (year of publication, country of origin, and the number of patients), patient characteristics (age), intervention characteristics (anesthesia induction drug, anesthesia maintenance drug), and data on the primary and secondary outcomes. When we found duplicate reports for the same trial, we analyzed data from the most complete data set. Disagreements were resolved by discussion with other reviewers.

The data are expressed as the mean ± standard deviation (SD). If the study provided the median and interquartile range instead of the mean and SD, we calculated the mean and SD using the method developed by McGrath et al. (18). For reports that only provided figures and where no exact data were available (despite contact with the authors), we extracted exact means and SDs from the figures using the program Engauge Digitizer 5.1 (Mitchell, Engauge Digitizer, http://digitizer.sourceforge.net), which can read exact values by digitizing data points from an image file after manually setting the coordinate axis.

### Risk-of-Bias Assessment

Two reviewers (JL Y and K K) independently assessed the risk of bias using the Cochrane Collaboration tool (19). We reviewed each trial and scored them as *high, low*, or *unclear* in terms of their risk involving the following domains: random sequence generation (selection bias), allocation concealment (selection bias), blinding of participants and personnel (performance bias), blinding of outcome assessment (detection bias), incomplete outcome data (attrition bias), selective reporting (reporting bias), and other bias types. Blinding of participants and personnel is generally not feasible in studies of this nature but we believe that the impact of blinding on study quality is not a particular concern, in this case, based on the particular nature of the study. Thus, we considered the low risk of bias trials to be those for which all key domains except the blinding of participants and personnel were considered low risk bias factors. All other trials were categorized as having unclear or a high risk of bias. Disagreements were resolved by discussion with a third reviewer (J L).

### Statistical Analysis

Review Manager (RevMan 5.3; Copenhagen: Nordic Cochrane Center Collaboration, 2014) was used for conducting the analysis. Differences are expressed as relative risks (RRs) with 95% confidence intervals (CIs) for dichotomous outcomes and standardized mean differences (SMDs) or weighted mean differences (WMDs) with 95% CIs for continuous outcomes. The SMD is the difference in mean outcome between groups divided by the standard deviation of the outcome variable among participants. Due to the variety of methods and units involved in the assessment of cytokine concentration levels among the different studies included, we adopted the SMD to standardize the study results to a uniform scale before combining them. Meta-analyses were performed using a random-effects model accounting for clinical heterogeneity. All analyses were performed based on the intention-to-treat principle. Statistical heterogeneity across trials was assessed by Cochran's Q test (with *p* < 0.1 indicating significance) and quantified by the *I*^2^ statistic (20). An *I*^2^ value exceeding 50% was considered to represent significant heterogeneity. Publication bias was not estimated because fewer than 10 studies were included.

## Results

### Trial Selection and Characteristics

**Figure 1:**
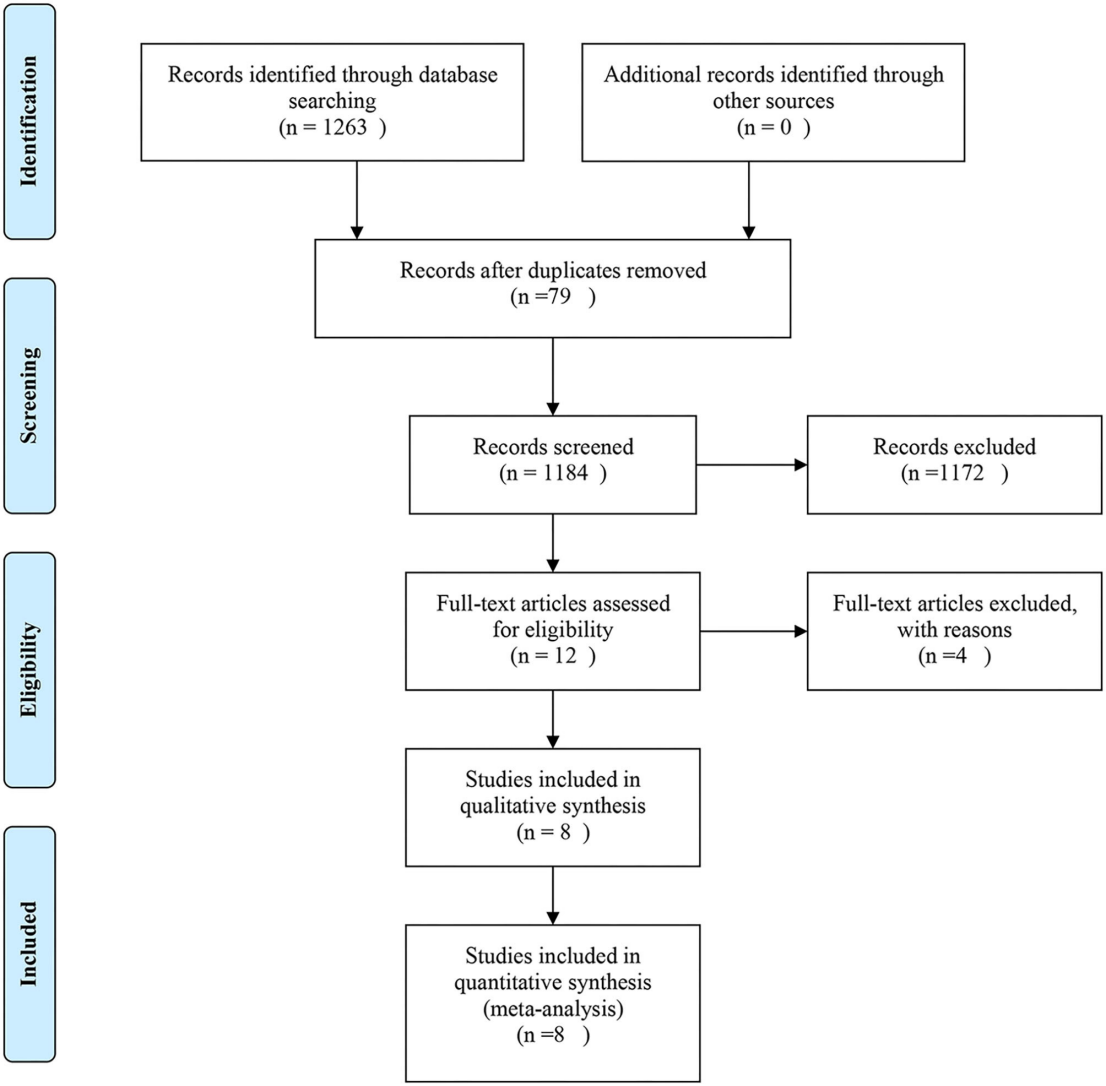
Flow diagram.

The Study Flow Diagram Is Shown in Figure 1. The initial search yielded 349 records. After removing duplicates and screening the titles and abstracts, 19 articles were deemed potentially eligible. After reviewing the full texts of the articles, eight trials (3, 7, 14, 15, 21–24) were included in the final analysis.

#### The Characteristics of the Included Trials

The eight included trials were published from 2009 to 2017, with sample sizes ranging from 36 to 174 subjects; in total, they included 488 subjects. In the sevoflurane group, the maintenance anesthetic was sevoflurane. In the propofol group, the maintenance anesthetic was propofol (Table 1).

**Table 1:** Main characteristics of randomized controlled trials included.

**References**	**Country**	**Year**	**NO. P/S**	**Age (years)**	**Information about operation**			**Intervention**	**Control**	**Outcomes**
					**Types of operation**	**NO.P**	**N0.S**			
Schilling et al. (3)	Germany	2011	21/21	P 64 (21–78) S 60 (24–83)^*a*^	Lobectomy/Pneumonectomy ectomy Atypical Pulmonary Resection	12/9	7/14	General anesthesia was induced with propofol and remifentanil Tracheal intubation was facilitated by cisatracurium and was maintained with sevoflurane remifentanil, cis-atracurium	General anesthesia was induced with propofol and remifentanil Tracheal intubation was facilitated by cisatracurium and was maintained with propofol remifentanil, cis-atracurium	Concentrations of IL-6, IL-8 IL-10, IL-1β, TNF-α in the ventilatory alveoli Concentrations of IL-6, IL-8 IL-10, IL-1β, TNF-α in the plasma, FIO_2_, PaO_2_, PaCO_2_, SaO_2_, MAP, CVP, CO H
De Conno et al. (7)	Switzerland	2009	27/27	P 58 ± 12 S 55 ± 15^*b*^	Thoracotomy/thoracoscopy	5/22	19/8	General anesthesia was induced with propofol Tracheal intubation was facilitated fentanyl and atracurium and was maintained with sevoflurane remifentanil, atracurium	General anesthesia was induced with propofol Tracheal intubation was facilitated fentanyl and atracurium and was maintained with propofol remifentanil, atracurium	Concentrations of IL-6, IL-8, TNF-α, IL-1β, MCP-1 in the non-ventilatory alveoli C-reactive Protein and Leukocyte Count in Blood Samples, patients with Pneumonia and Atelectasis
de la Gala et al. (14)	Spain	2017	88/86	P 64.5 (19–85) S 62.4 (25–88)^*c*^	pneumonectomy/bil-obectomy/Lobectomy-my/Segmentectomy	2/3/39/44	4/2/40/40	General anesthesia was induced with propofol, fentanyl, and rocuronium and was maintained with sevoflura	General anesthesia was induced with propofol, fentanyl, and rocuronium and was maintained with propofol	Concentrations of IL-6, IL-8 IL-10, IL-2, IL-1, IL-4, IL-7, TNF-α in the ventilatory alveoli; Concentrations of IL-6, IL-8, TNF-α, IL-10, IL-2, IL-1, IL-4, IL-7 in the non-ventilatory alveoli; Concentrations of IL-6, IL-10 IL-8, TNF-α, IL-2, IL-1, IL-4, IL-7 in the plasma, FEV1/FVC, MAP HR PaO_2_/FiO_2_, Patients with 0–1–2–>2PPCs
Jin et al. (15)	China	2013	20/20	P 62 ± 11 S 59 ± 13^*b*^	Elective thoracotomy lobectomy	20	20	General anesthesia was induced with sevoflurane, fentanyl, and rocuronium and was maintained with sevoflurane fentanyl, rocuronium	General anesthesia was induced with propofol, sufentanil, and rocuronium and was maintained with propofol fentanyl, rocuronium	Concentrations of IL-6, IL-10, and TNF-α, PA-aDO_2_, RI, Qs/Qt in the plasma PA-aDO_2_ RI Qs/Qt Cdyn
Sugasawa et al. (21)	Japan	2011	20/20	P 61.7 ± 13.5 S 62.9 ± 13.8^*b*^	Lobectomy/partial resection	13/7	14/6	General anesthesia was induced with sevoflurane, remifentanil Tracheal intubation was facilitated by rocuronium and was maintained with sevoflurane remifentanil	General anesthesia was induced with propofol, remifentanil, Tracheal intubation was facilitated by rocuronium and was maintained with sevoflurane remifentanil, rocuronium	Concentrations of IL-6, IL-8 IL-1β in the ventilatory alveoli Concentrations of IL-6, IL-8, IL-1β in the non-ventilatory alveoli
Hammouda et al. (22)	Egypt	2013	20/20	P 52.9 ± 9.8 S 54.5 ± 12.4^*b*^	Elective lung resection surgery through thoracotomy	20	20	General anesthesia was induced with propofol, fentanyl Tracheal intubation was facilitated by cisatracurium and was maintained with sevoflurane	General anesthesia was induced with propofol, fentanyl Tracheal intubation was facilitated by cisatracurium and was maintained with propofol	Concentrations of IL-6, TNF-α in the non-ventilatory alveoli. Concentrations of IL-6, TNF-α in the plasma pH, PaO2, PaCo2, HCO
Tian et al. (23)	China	2017	31/31	P 68.3 ± 13.5 S 65.5 ± 16.2^*b*^	Pulmonary lobectomy	31	31	General anesthesia was induced with sevoflurane, and was maintained with sevoflurane	General anesthesia was induced with propofol, and was maintained with propofol	Concentrations of IL-6, IL-10, MMP-9, PA-aDO_2_,RI,Qs/Qt in the plasma. The extubation time, eye opening time and response time of patients after operation
Potočnik et al. (24)	Slovenia	2014	19/17	P 60.9 ± 9.4 S 52.7 ± 14.6^*b*^	Elective open lobectomy	19	17	General anesthesia was induced with sevoflurane, remifentanil vecuronium and was maintained with sevoflurane	General anesthesia was induced with propofol, remifentanil vecuronium and was maintained with propofol	Concentrations of IL-6, IL-8, IL-10, PaO_2_/FiO_2_ in the plasma, patients with pneumonia

*P, propofol; S, sevoflurane; I, intervention group (sevoflurane); II, control group (propofol)*.

a *Data are given as the median (range)*.

b *Data are given as mean ± SD*.

c *Data are expressed as mean (range)*.

#### Details of the Risk of Bias

**Figure 2:**
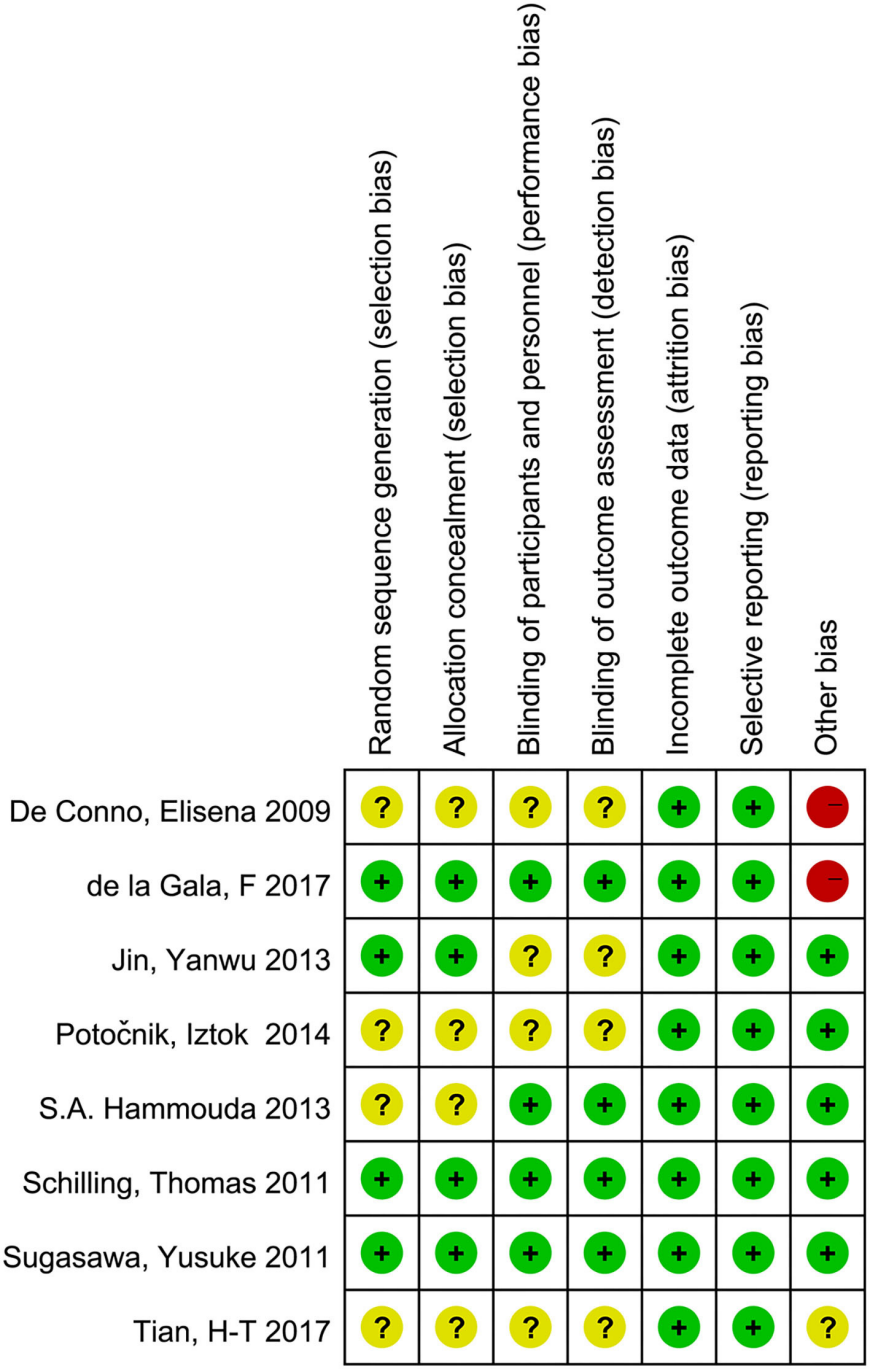
Risk of bias summary.

Overall, two trials (3, 21) were categorized as having a low risk of bias, four trials (15, 22–24) had an unclear risk of bias, and two trials (7, 14) were categorized as having a high risk of bias (Figure 2).

### Primary Outcome

**Figure 3:**
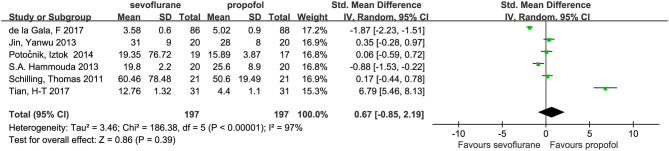
Forest plot for the concentrations of systemic interleukin-6.

**Figure 4:**
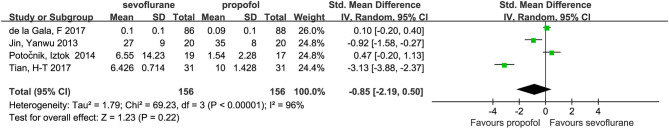
Forest plot for the concentrations of systemic interleukin-10.

**Figure 5:**
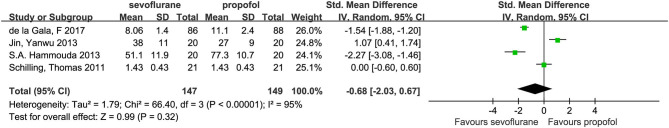
Forest plot for the concentrations of systemic tumor necrosis factor α.

The pooled results from the random-effects model combining the SMDs for systemic IL-6, IL-10, and TNF-α are shown in Figures 3–**5**, respectively.

#### Interleukin-6 in the Plasma

Six studies (3, 14, 15, 22–24) with a total of 394 patients provided the plasma concentrations of IL-6. No significant difference was found in these concentrations between the groups (SMD, 0.67; 95% CI, −0.85 to 2.19; Figure 3).

#### Interleukin-10 in the Plasma

Four studies (14, 15, 23, 24) with a total number of 312 patients provided the plasma concentrations of IL-10. No significant difference in these concentrations was found between the groups (SMD, −0.85; 95% CI, −2.19 to 0.50; Figure 4).

#### Tumor Necrosis Factor α in the Plasma

Four studies (3, 14, 15, 22) with a total number of 296 patients provided the plasma concentrations of TNF-α. No significant difference in these concentrations was found between the groups (SMD, −0.68; 95% CI, −2.03 to 0.67; Figure 5).

## Secondary Outcomes

### Interleukin-6 in the Dependent Ventilated Lung

**Figure 6:**
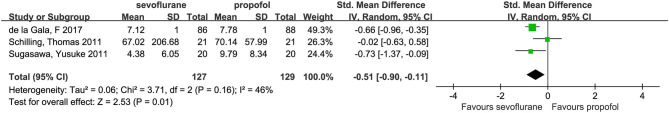
Forest plot for the concentration of dependent alveolar interleukin-6.

Three studies (3, 14, 21) investigated dependent alveolar concentrations of IL-6. In these studies, the concentrations of alveolar IL-6 were lower in the sevoflurane group than in the propofol group (SMD, −0.51; 95% CI, −0.90 to −0.11; *p* = 0.01; Figure 6).

### Interleukin-8 in the Dependent Ventilated Lung

**Figure 7:**
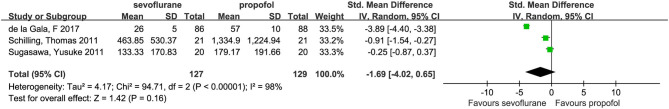
Forest plot for the concentration of dependent alveolar interleukin-8.

Three studies investigated alveolar concentrations of IL-8 (3, 14, 21); their pooled results (SMD, −1.69; 95% CI, −4.02 to 0.65; *p* = 0.16; Figure 7) indicated a declining but statistically insignificant trend for IL-8 levels in the sevoflurane group compared with the propofol group. However, we detected a marked degree of heterogeneity between the studies (*I*^2^ = 98%). To further explore the potential causes of this high heterogeneity, we performed a sensitivity analysis by omitting one study (14) at a time from our pooled data synthesis. The result of this analysis indicated that the difference between the two groups was not statistically significant; however, the heterogeneity was reduced, and the SMD (95% CI) was −0.57 (−1.22 to 0.07; *I*^2^ = 52%; *p* = 0.08).

### Interleukin-6 in the Independent Ventilated Lung

**Figure 8:**
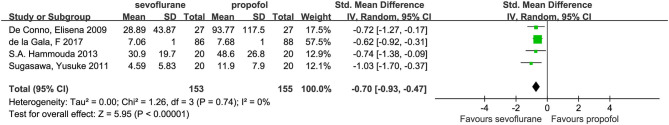
Forest plot for the concentration of independent alveolar interleukin-6.

Four studies (7, 14, 21, 22) investigated the independent alveolar concentrations of IL-6, among which concentrations were lower in the sevoflurane group compared with the propofol group (SMD, −0.70; 95% CI, −0.93 to −0.47; *p* < 0.01; Figure 8).

### Interleukin-8 in the Independent Ventilated Lung

**Figure 9:**
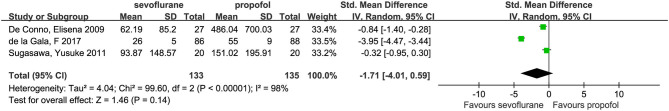
Forest plot for the concentration of independent alveolar interleukin-8.

No statistical heterogeneity was observed across trials (*I*^2^ = 0%). Three studies (7, 14, 21) investigated independent alveolar concentrations of IL-8, and the pooled results (SMD, −1.71; 95% CI, −4.01 to 0.59; *p* = 0.14; Figure 9) indicated a declining but statistically insignificant trend for IL-8 levels in the sevoflurane group compared with the propofol group. However, a marked degree of heterogeneity between the studies (*I*^2^ = 98%) was detected. To further explore the potential causes of this high heterogeneity, we performed a sensitivity analysis by omitting one study at a time from our pooled data synthesis. The result of this analysis was that the difference between the two groups was not statistically significant; however, the heterogeneity was reduced, and the mean difference (95% CI) was −0.60 (−1.10 to −0.10); s, *I*^2^ = 31%; *p* = 0.02.

### Tumor Necrosis Factor α in the Independent Ventilated Lung

**Figure 10:**
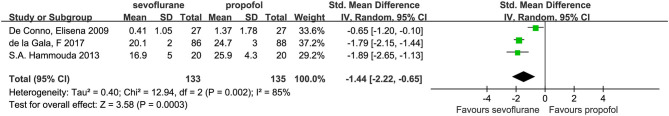
Forest plot for the concentration of independent alveolar tumor necrosis factor α.

Three studies (7, 14, 22) investigated independent alveolar concentrations of TNF-α, among which the concentrations were lower in the sevoflurane group compared with the propofol group (SMD, −1.44; 95% CI, −2.22 to −0.65; *p* < 0.001; Figure 10). However, we detected a marked degree of heterogeneity between the studies (*I*^2^ = 85%). To further explore the potential causes of this high heterogeneity, we performed a sensitivity analysis by omitting one study at a time from our pooled data synthesis. The result of this analysis was that the difference between the two groups was not statistically significant; however, the heterogeneity was reduced, and the mean difference (95% CI) was −1.81; 95% CI, −2.13 to −1.49; *I*^2^ = 0%; *p* < 0.001.

### Sensitivity Analysis

The results of this study showed high heterogeneity when comparing the differences in IL-8 between the two anesthetic drugs in the dependent lung. We conducted a sensitivity analysis on the results and found that the heterogeneity derived from a study by de la Gala et al. (14). Following a comparison, heterogeneity was found to have been primarily the result of the following. The main purpose of that study had been to investigate the effect of sevoflurane and propofol on the incidence of pulmonary complications. The inflammatory response was only a secondary outcome of this study and, as such, additional efforts are needed to further verify these results.

Only two studies discussed the dependent alveolar IL-10 and TNF-α concentrations, independent alveolar IL-10 concentrations, and plasma IL-8 concentrations. Accordingly, we did not analyze these studies. When analyzing the TNF-α levels in the dependent lung, the heterogeneity was found to be high. Following the sensitivity analysis, the heterogeneity was found to have been derived from the study of De Conno Elisena, in 2009. Although the heterogeneity was reduced to 2% following the removal of this study, there were only two studies left; therefore, the results obtained required further verification.

In the process of comparing the levels of systemic inflammatory cytokines, the heterogeneity of IL-6, IL-10, and TNF-α was found to be relatively large; therefore, we carried out a sensitivity analysis. After systemically removing the studies, we discovered the heterogeneity changes were relatively small, thus indicating that the results were stable and, as such, had a level of credibility. The sources of heterogeneity may have been related to the diverse range of study countries, different participant ages, different inflammatory factor measurement personnel, and variable alveolar lavage fluid and blood sampling times.

## Discussion

The objective of this meta-analysis, which included eight articles (488 patients), was to evaluate the effects of sevoflurane and propofol on inflammatory responses in patients undergoing lung resection with OLV. The results of this study showed no difference in systemic inflammatory factors (IL-6, IL-10, TNF-α) between the two groups. Compared with propofol, IL-6 in the dependent lung and IL-8 in the independent lung were decreased by sevoflurane.

For data expressed in the form of a median and interquartile range, we used the method provided by McGrath to convert the data into mean ± SD for analysis (18). During the analysis, data with obvious errors and with fewer than three references were discarded; excluded studies included those that investigated BAL levels of IL-10 and TNF-α in the dependent ventilated lung and BAL levels of IL-10 and TNF-α in the independent ventilated lung.

The inflammatory cytokine response is an important mechanism of the pulmonary inflammatory response induced by one-lung ventilation, and the mechanism of the inflammatory response between the dependent lung and independent lung is different during OLV. Mechanical ventilation of the dependent lung causes damage to the alveolar epithelium and releases pro-inflammatory cytokines, which are also released by collapse and re-inflation of the independent lung (25, 26). Therefore, the inflammatory response of the dependent and independent lung was analyzed separately in this study. A previous meta-analysis (27) showed that inhaled anesthetics (sevoflurane or desflurane) could reduce the intraoperative inflammatory response when compared with propofol; however, two surgical types were included in this particular study, which increased the heterogeneity of its results. In the present study, it is concluded that compared with propofol, sevoflurane can reduce the local inflammatory response of the ventilated and non-ventilated lung, which is supported by an existing study (27). However, the current study found no difference in the systemic inflammatory response between sevoflurane and propofol. Sevoflurane was found to play an anti-inflammatory role by activating the lung gamma-aminobutyric acid (A) signaling pathway, reducing alveolar cytokines and ultimately inhibiting pulmonary inflammation, thereby protecting the lungs from secondary attacks (28). One study showed that sevoflurane could not only change the expression of inflammatory mediators in alveolar epithelial cells but also affect the accumulation of neutrophils, thereby changing the inflammatory response (29, 30).

The above results are consistent with the conclusion of our meta-analysis, i.e., sevoflurane could reduce the inflammatory response of local alveoli. Our study showed that, compared with propofol, sevoflurane had an inhibitory effect on both dependent and independent alveolar inflammatory responses. The specific mechanism in this context is unclear, however, specifically, whether the effect on the independent pulmonary inflammatory response is direct or indirect still requires confirmation through additional research. Video-assisted thoracic surgery (VATS) has been developed greatly for patients with lung cancer. The previous study showed VATS induced less inflammatory response than open thoracic surgery (31). However, whether less inflammatory response following VATS contributes to improved clinical outcomes is unknown and more studies are needed to confirm the benefits of VATS for patients.

The results of this study showed that there was high heterogeneity when comparing IL-8 differences between the two anesthetic drugs in the dependent lung. We conducted a sensitivity analysis on the results and found that heterogeneity was derived from the study by de la Gala et al. (14).

In the process of comparing the levels of systemic inflammatory cytokines, it was found that the heterogeneity of IL-6, IL-10, and TNF-α was relatively large. As a result, we carried out a sensitivity analysis. After removing the studies systemically, we found that the heterogeneity changes were relatively small, indicating that the results were stable and, as such, had a level of credibility. The sources of heterogeneity may have been related to the different study countries, different participant ages, different inflammatory factor measurement personnel, and different alveolar lavage fluid measurements.

The present study includes some limitations. The eight included papers were studied in different regions including Asia (China), Africa (Egypt), and Europe (Germany, Spain, Switzerland, and Slovenia). Therefore, the results do not apply to patients in South America, North America, or Oceania.

In conclusion, there was no difference in the systemic inflammatory response between sevoflurane and propofol. However, compared with propofol, sevoflurane can reduce the local alveolar inflammatory response.

## Data Availability Statement

The original contributions presented in the study are included in the article/Supplementary Material, further inquiries can be directed to the corresponding author/s.

## Author Contributions

J-LY and J-QZ: conception and design of the research. J-LY and XK: acquisition of data. KK and WZ: analysis and interpretation of the data. BL and JL: statistical analysis. WZ: obtaining financing. KK and BL: writing of the manuscript. M-RM, XK, and WZ: critical revision of the manuscript for intellectual content.

## Conflict of Interest

The authors declare that the research was conducted in the absence of any commercial or financial relationships that could be construed as a potential conflict of interest.

## References

[1] Okamoto K Hayashi K Kaku R Kawaguchi Y Oshio Y Hanaoka J. Impact of fractional exhaled nitric oxide on the outcomes of lung resection surgery: a prospective study. J Thorac Dis. (2020) 12:2663–71. 10.21037/jtd.2020.03.18 32642174 PMC7330331

[2] Schilling T Kozian A Huth C Bühling F Kretzschmar M Welte T. The pulmonary immune effects of mechanical ventilation in patients undergoing thoracic surgery. Anesth Analg. (2005) 101:957–65. 10.1213/01.ane.0000172112.02902.77 16192502

[3] Schilling T Kozian A Senturk M Huth C Reinhold A Hedenstierna G. Effects of volatile and intravenous anesthesia on the alveolar and systemic inflammatory response in thoracic surgical patients. Anesthesiology. (2011) 115:65–74. 10.1097/ALN.0b013e318214b9de 21399490

[4] Cantor H Boyse EA. Functional subclasses of T lymphocytes bearing different Ly antigens. II Cooperation between subclasses of Ly+ cells in the generation of killer activity. J Exp Med. (1975) 141:1390–9. 10.1084/jem.141.6.1390 1092799 PMC2189854

[5] Lohser J. Evidence-based management of one-lung ventilation. Anesthesiol Clin. (2008) 26:241–72. 10.1016/j.anclin.2008.01.011 18456211

[6] Tekinbas C Ulusoy H Yulug E Erol MM Alver A Yenilmez E. One-lung ventilation: for how long? J Thorac Cardiovasc Surg. (2007) 134:405–10. 10.1016/j.jtcvs.2007.05.003 17662780

[7] De Conno E Steurer MP Wittlinger M Zalunardo MP Weder W Schneiter D. Anesthetic-induced improvement of the inflammatory response to one-lung ventilation. Anesthesiology. (2009) 110:1316–26. 10.1097/ALN.0b013e3181a10731 19417610

[8] Lee JJ Kim GH Kim JA Yang M Ahn HJ Sim WS. Comparison of pulmonary morbidity using sevoflurane or propofol-remifentanil anesthesia in an Ivor Lewis operation. J Cardiothorac Vasc Anesth. (2012) 26:857–62. 10.1053/j.jvca.2012.01.015 22381051

[9] Craig SR Leaver HA Yap PL Pugh GC Walker WS. Acute phase responses following minimal access and conventional thoracic surgery. Eur J Cardiothorac Surg. (2001) 20:455–63. 10.1016/S1010-7940(01)00841-7 11509263

[10] Sugasawa Y Yamaguchi K Kumakura S Murakami T Kugimiya T Suzuki K. The effect of one-lung ventilation upon pulmonary inflammatory responses during lung resection. J Anesth. (2011) 25:170–7. 10.1007/s00540-011-1100-0 21301891

[11] Broccard AF Hotchkiss JR Suzuki S Olson D Marini JJ. Effects of mean airway pressure and tidal excursion on lung injury induced by mechanical ventilation in an isolated perfused rabbit lung model. Crit Care Med. (1999) 27:1533–41. 10.1097/00003246-199908000-00022 10470761

[12] Slutsky AS. Lung injury caused by mechanical ventilation. Chest. (1999) 116(1 Suppl):9S−15S. 10.1378/chest.116.suppl_1.9S-a 10424561

[13] Baudouin SV. Lung injury after thoracotomy. Br J Anaesth. (2003) 91:132–42. 10.1093/bja/aeg083 12821572

[14] de la Gala F Piñeiro P Reyes A Vara E Olmedilla L Cruz P. Postoperative pulmonary complications, pulmonary and systemic inflammatory responses after lung resection surgery with prolonged one-lung ventilation. Randomized controlled trial comparing intravenous and inhalational anaesthesia. Br J Anaesth. (2017) 119:655–63. 10.1093/bja/aex230 29121283

[15] Jin Y Zhao X Li H Wang Z Wang D. Effects of sevoflurane and propofol on the inflammatory response and pulmonary function of perioperative patients with one-lung ventilation. Exp Ther Med. (2013) 6:781–5. 10.3892/etm.2013.1194 24137265 PMC3786802

[16] Cumpston M Li T Page MJ Chandler J Welch VA Higgins JP. Updated guidance for trusted systematic reviews: a new edition of the cochrane handbook for systematic reviews of interventions. Cochrane Database Syst Rev. (2019) 10:ED000142. 10.1002/14651858.ED000142 31643080 PMC10284251

[17] Moher D Liberati A Tetzlaff J Altman DG; PRISMA Group. Preferred reporting items for systematic reviews and meta-analyses: the PRISMA statement. PLoS Med. (2009) 6:e1000097. 10.1371/journal.pmed.1000097 19621072 PMC2707599

[18] McGrath S Zhao X Steele R Thombs BD Benedetti A; DEPRESsion Screening Data (DEPRESSD) Collaboration. Estimating the sample mean and standard deviation from commonly reported quantiles in meta-analysis. Stat Methods Med Res. (2020) 962280219889080. 10.1177/0962280219889080 PMC7390706 32292115

[19] Higgins JP Altman DG Gøtzsche PC Jüni P Moher D Oxman AD. The Cochrane Collaboration's tool for assessing risk of bias in randomised trials. BMJ. (2011) 343:d5928. 10.1136/bmj.d5928 22008217 PMC3196245

[20] Houston M. Ireland leads the way for Europe in banning smoking in the workplace. BMJ. (2003) 327:522. 10.1136/bmj.327.7414.522 12958102 PMC192844

[21] Sugasawa Y Yamaguchi K Kumakura S Murakami T Suzuki K Nagaoka I. Effects of sevoflurane and propofol on pulmonary inflammatory responses during lung resection. J Anesth. (2012) 26:62–9. 10.1007/s00540-011-1244-y 21979104

[22] Hammouda SA Abd Rabbih AA Alganady AA Ghoneim TA Elsawy MM Youssif SA. Immunomodulatory effect of propofol versus sevoflurane in patients undergoing thoracic surgery using one lung ventilation technique. Egypt J Chest Dis Tuberc. (2013) 62:731–43. 10.1016/j.ejcdt.2013.08.005

[23] Tian HT Duan XH Yang YF Wang Y Bai QL Zhang X. Effects of propofol or sevoflurane anesthesia on the perioperative inflammatory response, pulmonary function and cognitive function in patients receiving lung cancer resection. Eur Rev Med Pharmacol Sci. (2017) 21:5515–22. 10.26355/eurrev_201712_13943 29243798

[24] Potočnik I Novak Janković V Šostarič M Jerin A Štupnik T Skitek M. Antiinflammatory effect of sevoflurane in open lung surgery with one-lung ventilation. Croat Med J. (2014) 55:628–37. 10.3325/cmj.2014.55.628 25559834 PMC4295075

[25] Tsai JA Lund M Lundell L Nilsson-Ekdahl K. One-lung ventilation during thoracoabdominal esophagectomy elicits complement activation. J Surg Res. (2009) 152:331–7. 10.1016/j.jss.2008.03.046 18708192

[26] Langlois-Karaga A Bues-Charbit M Balansard G Charpin D Vervloet D Sutra JP. Variations spatiales et temporelles des quantités de pollen de Cupressacées-Taxacées recueillies à Marseille [Spatial and temporal variations of the quantities of pollen from Cupressaceae-Taxaceae collected in Marseilles]. Allerg Immunol. (1992) 24:213–15. 1497797

[27] Sun B Wang J Bo L Zang Y Gu H Li J. Effects of volatile vs. propofol-based intravenous anesthetics on the alveolar inflammatory responses to one-lung ventilation: a meta-analysis of randomized controlled trials. J Anesth. (2015) 29:570–9. 10.1007/s00540-015-1987-y 25716536

[28] Fortis S Spieth PM Lu WY Parotto M Haitsma JJ Slutsky AS. Effects of anesthetic regimes on inflammatory responses in a rat model of acute lung injury. Intensive Care Med. (2012) 38:1548–55. 10.1007/s00134-012-2610-4 22711173 PMC4896809

[29] Suter D Spahn DR Blumenthal S Reyes L Booy C Z'graggen BR. The immunomodulatory effect of sevoflurane in endotoxin-injured alveolar epithelial cells. Anesth Analg. (2007) 104:638–45. 10.1213/01.ane.0000255046.06058.58 17312223

[30] Torres MF Porfírio GJ Carvalho AP Riera R. Non-invasive positive pressure ventilation for prevention of complications after pulmonary resection in lung cancer patients. Cochrane Database Syst Rev. (2019) 3:CD010355. 10.1002/14651858.CD010355.pub3 30840317 PMC6402531

[31] Ng CS Lau KK. Surgical trauma and immune functional changes following major lung resection. Indian J Surg. (2015) 77:49–54. 10.1007/s12262-013-0957-6 25829712 PMC4376837

