# Supplementary material for: The Effects of Sevoflurane vs. Propofol for Inflammatory Responses in Patients Undergoing Lung Resection: A Meta-Analysis of Randomized Controlled Trials
Source: Front Surg. 2021 Jul 2;8:692734. doi: 10.3389/fsurg.2021.692734 (PMC8282814; doi:10.3389/fsurg.2021.692734)
Supplement: Supplementary file 1 [file Table_1.DOC]

The title:Effects of sevoflurane versus propofol as maintenance anesthetics for inflammatory responses and pulmonary complications in patients undergoing lung resection: meta-analysis of randomized controlled trials

The first author:Jingli Yuan

| cochrane |  |
| --- | --- |
| #1 | MeSH descriptor: [One-Lung Ventilation] explode all trees |
| #2 | (ventilation, one lung):ti,ab,kw OR (single lung ventilation):ti,ab,kw OR (single lung ventilation):ti,ab,kw OR (single lung ventilations):ti,ab,kw OR (ventilation, single lung):ti,ab,kw |
|
| #3 | (ventilations, single lung):ti,ab,kw OR (single lung ventilation):ti,ab,kw OR (single lung ventilation):ti,ab,kw OR (single lung ventilations):ti,ab,kw OR (ventilation, single lung):ti,ab,kw |
|
| #4 | #2 OR #3 |
| #5 | #1 OR #4 |
| #6 | MeSH descriptor: [Sevoflurane] explode all trees |
| #7 | (anesthetics,inhalation):ti,ab,kw OR (Inhalation,anesthesia):ti,ab,kw OR (Inhalational,anesthesia):ti,ab,kw OR (Inhaled,anesthesia):ti,ab,kw OR (ventilation, single lung):ti,ab,kw |
|
| #8 | #6 OR #7 |
| #9 | MeSH descriptor: [Propofol] explode all trees |
| #10 | (intravenous,anesthesia):ti,ab,kw OR (intravenous,anesthetics):ti,ab,kw OR (anesthetics,intravenous):ti,ab,kw OR (intravenous,anesthetic agent):ti,ab,kw OR (ventilation, single lung):ti,ab,kw |
|
| #11 | #9 OR #10 |
| #12 | #5 AND #8 AND #11 |

| wos |  |
| --- | --- |
|  | TOPIC: (one lung ventilation) AND TOPIC: (propofol) AND TOPIC: (sevoflurane) |

| pubmed |  |
| --- | --- |
| #1 | Search: "One-Lung Ventilation"[Mesh] Sort by: Most Recent |
| #2 | Search: (((((ventilation, one lung[Title/Abstract]) OR (single lung ventilation[Title/Abstract])) OR (single lung ventilation[Title/Abstract])) OR (single lung ventilations[Title/Abstract])) OR (ventilation, single lung[Title/Abstract])) OR (ventilations, single lung[Title/Abstract]) |
|
|
| #3 | #1 OR #2 |
| #4 | Search: "Sevoflurane"[Mesh] Sort by: Most Recent |
| #5 | Search: (((anesthetics,inhalation[Title/Abstract]) OR (Inhalation,anesthesia[Title/Abstract])) OR (Inhalational,anesthesia[Title/Abstract])) OR (Inhaled,anesthesia[Title/Abstract]) |
| #6 | #4 OR #5 |
| #7 | Search: "Propofol"[Mesh] Sort by: Most Recent |
| #8 | Search: (((intravenous,anesthesia[Title/Abstract]) OR (intravenous,anesthetics[Title/Abstract])) OR (anesthetics,intravenous[Title/Abstract])) OR (intravenous,anesthetic agent[Title/Abstract]) |
| #9 | #7 OR #8 |
| #10 | #3 AND #6 AND #9 |

| embase |  |
| --- | --- |
| #1 | ('one lung ventilation'/exp OR 'one lung ventilation' OR 'one-lung ventilation' OR 'one-side lung ventilation' OR 'one-sided lung ventilation' OR 'single lung ventilation') AND 'sevoflurane'/exp AND 'propofol'/exp AND ('inflammation'/exp OR 'inflammation reaction' OR 'inflammation response' OR 'inflammatory process' OR 'inflammatory reaction' OR 'inflammatory response' OR 'reaction, inflammation' OR 'response, inflammatory') |
|
|
|
|
|
